# Impacts of Ultrasonogram-Guided, Intra-fascial, Autologous, Activated Platelet-Rich Plasma Injection in Chronic Plantar Fasciitis: A Quasi-experimental Study

**DOI:** 10.7759/cureus.34659

**Published:** 2023-02-05

**Authors:** Md. Nuruzzaman Khandaker, Md. Mahbubul Islam, Md. Ali Emran, Badrunnesa Ahmed, Farzana Khan Shoma, Moshiur R Khasru, Saosun Binta Rob, Sarjana Yeasmin, Redoy Ranjan

**Affiliations:** 1 Physical Medicine and Rehabilitation, Bangabandhu Sheikh Mujib Medical University, Dhaka, BGD; 2 Physical Medicine and Rehabilitation, Manikganj Sadar Hospital, Manikganj, BGD; 3 Surgical Science, The University of Edinburgh, e-Tutor, Edinburgh, GBR; 4 Institute of Cardiovascular Research, Royal Holloway University of London, London, GBR; 5 Cardiac Surgery, Bangabandhu Sheikh Mujib Medical University, Dhaka, BGD

**Keywords:** sports physiotherapy, first step pain, foot pain, chronic plantar fasciitis, platelet-rich plasma, plantar fasciitis

## Abstract

Background

Plantar fasciitis is the most common cause of foot pain. Patients with plantar fasciitis typically present with ‘first step pain,’ which tends to decrease with activity and worse with heavy use. This study determines the effect of ultrasound-guided, single-dose, platelet-rich plasma (PRP) injection in patients with chronic plantar fasciitis.

Materials and methods

It was a quasi-experimental trial carried out in the Department of Physical Medicine and Rehabilitation, Bangabandhu Sheikh Mujib Medical University (BSMMU), Dhaka, Bangladesh, from March 2019 to March 2022. A total of 148 patients diagnosed with chronic plantar fasciitis were selected as samples. A total of 75 patients were allocated to group A (intra-lesional injection of autologous PRP with conservative management) and 73 patients to Group B (only conservative management). Both groups of patients were allocated to conservative management with exercises, shoe modification, activities of daily living (ADLs) instruction, and oral paracetamol.

Results

This study shows that in group A, the mean visual analog scale (VAS) score significantly reduced to 1.47±0.51 after six months of single-dose PRP injection (p<0.001). In group B, the VAS score also decreased substantially after conservative treatment. Though in groups A and B, pain reduction was significant, in group A, the pain was decreased more compared to group B and statistically significant differences were found between the two groups at the 12th week and 24th week. The foot function index (FFI) scores decreased significantly in group A after a single dose PRP injection, compared with group B treated with conventional therapy. In group A, FFI scores decreased from 49.09±5.72 to 7.67±3.41. The study revealed a significant difference between study groups in the 12th week and 24th week regarding FFI scores.

Conclusion

Ultrasound-guided intra-lesional autologous PRP Injection is safe and effective and recommended in patients with chronic plantar fasciitis, especially in recalcitrant cases after the failure of conservative treatment and corticosteroid injection.

## Introduction

The plantar fascia is a fibrous aponeurosis that originates from medial calcaneal tuberosity and extends distally, becoming broader and thinner splitting into five bands. Each band then splits into a superficial and deep layer to insert into the transverse tarsal ligament, volar plate, flexor sheath, and periosteum of the proximal phalanges of the toes [1]. Heel pain is a common complaint in foot and ankle practice. Plantar fasciitis (PF) is the most common cause of heel pain [2]. The peak incidence may occur between the ages of 40 to 60 years [3].

Predisposing factors for plantar fasciitis include reduced ankle dorsiflexion, obesity, work-related weight bearing, and prolonged standing, walking, or running on uneven and hard surfaces [4]. In plantar fasciitis, there are microtears at the junction of bone and fascia. These tears can be due to sudden weight gain, long-distance running, flat shoes, improper shoe inserts, tight Achilles tendon, etc. These microtears are the root cause of the problem. Healing of these microtears is associated with pain relief [5].

Various types of treatments have been advocated for treating this condition like non-steroidal anti-inflammatory drugs (NSAIDs), night splints, ice packs, heat, massage, shoe modification, plantar fascia stretching exercise, local corticosteroid injections, surgery, etc. [3]. Several studies have used platelet-rich plasma (PRP) to manage soft tissue and bone injuries. Platelet-rich plasma has recently been used for cartilage regeneration, chronic enthesopathy like tennis elbow, plantar fasciitis, and sports medicine [6].

PRP is a plasma fraction of autologous blood having a high concentration of platelets compared to the baseline value [5-7]. The platelet α granules are rich in growth factors that play an essential role in tissue healing such as endothelial growth factor, transforming growth factor-β, and platelet-derived growth factor. PRP is used in various surgical fields to enhance bone and soft-tissue healing by placing supraphysiological concentrations of autologous platelets at the site of tissue damage [7].

The use of PRP in treating plantar fasciitis is a relatively recent and evolving concept. Injection of PRP is safe and doesn’t affect the biomechanical function of the foot [8]. Therefore, the present study aimed to determine the effects of single-dose, intra-fascial, autologous, activated PRP injection under ultrasonogram guidance in patients with chronic plantar fasciitis.

## Materials and methods

It was a quasi-experimental study carried out in the Department of Physical Medicine and Rehabilitation, Bangabandhu Sheikh Mujib Medical University (BSMMU), Bangladesh, from March 2019 to March 2022. A total of 150 patients were selected as samples that were diagnosed with plantar fasciitis through clinical and sonography findings. The selected patients were allocated to group A and group B according to their choice. In group A, the patients were given intra-lesional injections of autologous PRP with conservative management, and in group B, only conservative management was given. Both groups of patients were allocated exercises, shoe modification, activities of daily living (ADLs) instruction, and oral paracetamol. Ethical clearance was taken from the Institutional Review Board (Ref. BSMMU/2019/5700), Bangabandhu Sheikh Mujib Medical University, Bangladesh.

Foot function index (FFI)

The FFI was developed to measure the impact of foot pathology on function in terms of pain, disability, and activity restriction [8].

Visual analog scale (VAS)

VAS is designed to measure the severity of symptoms in individual patients and use this to achieve a rapid (statistically measurable and reproducible) classification of symptom severity and disease control [9].

Intra-lesional autologous PRP injection technique

The patient was placed in a supine position with the knee fully extended. First, the sites of maximal tenderness were located by thumb pressure over the origin of the plantar fascia, which was reconfirmed and marked with a skin marker under an ultrasound scan. With all aseptic precautions, the posterior tibial nerve and particularly the medial calcaneal branch were injected with lidocaine hydrochloride 1% finger breadth behind and distal to the medial malleolus. Then, 5 ml of local anesthesia was injected, and a gentle thumb massage was applied for 30 seconds over the injection zone. Once adequate local anesthesia was documented, the marked point of tenderness was penetrated with a 23-gauge needle until the underlying periosteum was found by touch. An end-feel experience with a gristly and crunchy texture was audibly and palpably noted as the needle was advanced. After contacting the periosteum, the needle was gently partially withdrawn and then advanced in a fan-like wheel (peppering) the area seven to 10 times. Next, 2.5 mL of the PRP is injected with a 23-gauge needle as this peppering maneuver is continued. The point of needle entry should be more medial than plantar [10]. As PRP is a biological product, so the average standard adult dose is 2.5 ml PRP was given to every patient in group A [11].

The patient rested for 10 minutes after the injection and was discharged after 30 minutes of observation. During discharge, the patient was advised a crepe bandage and ice compression for 10 mins three times daily for the first three days. No aggressive running or jumping activities were allowed for two weeks. Night splinting was used for comfort. Gradual return to activities was permitted after three weeks [10]. Only paracetamol (dosage 500 mg tds for the first seven days) was prescribed and was allowed on demand in case of discomfort and pain. The drugs were used by only one pharmaceutical company to avoid efficacy differences. All patients were requested to be 24 hours analgesia-free before baseline and every follow-up measurement. All patients were advised to avoid non-steroidal anti-inflammatory drugs (NSAIDs) for six months, an antiplatelet medicine potentially antagonizing our activated platelet-rich plasma injection activity. However, paracetamol was only allowed for severe pain with a dosage of <2000 mg/day [12].

Conservative management

Exercises

(i) Achilles stretching exercise: The patients were instructed to perform the stretches three times a day, three repetitions for each stretch, and hold each stretch for 30 seconds at a time [13].

(ii) Strengthening exercise is initiated after two weeks of injection. Intrinsic foot muscle strengthening exercise. The patients were instructed to perform the exercise three times a day, 10 repetitions, and hold each position for 10 seconds at a time [14]. Shoe modification: Silicon heel cushion, use shock absorbing shoe. Instructions for activities of daily living (ADLs) were prescribed for all patients: use shoes with a soft sole, avoid prolonged standing, avoid bare feet during walking, reduce weight in overweight patients, and use silicon heel cushions.

Follow-up procedure

Data were collected through a face-to-face interview with the help of a semi-structured questionnaire. All items were rated using a visual analog scale. A total foot function score is derived by calculating the average of the three sub-scale scores. To eliminate the decimal point, the score is multiplied by 100. Therefore, sub-scale scores range from 0 to 100, with higher scores indicating more significant impairment. A follow-up visit was given to each patient in the 4th week, 12th week, and 24th week. During each follow-up visit, the data information sheet recorded any improvement and appearance of side effects. The data collected from the patients were analyzed. After completion of data collection, the data were checked, coded, entered, and analyzed in a computer to develop an Excel file (Microsoft Corporation, Redmond, WA).

Statistical analysis

The statistical analysis was conducted using SPSS (Statistical Package for the Social Sciences) version 25 statistical software. The findings of the study were presented by frequency percentage in tables. Means and standard deviations for continuous variables and frequency distributions for categorical variables were used to describe the characteristics of the total sample. Associations of categorical data were assessed using the chi-square test. Associations of continuous data were evaluated using the student’s t-test and paired t-test, and all p-values were two-sided where p<0.05 was considered significant.

## Results

A total of 148 consecutive patients were evaluated, and preoperative sociodemographic variables were statistically insignificant between study populations (Table 1). The foot function index (FFI) sub-scale distribution between the two groups is shown in Table 2, and no significant differences were observed among the study population.

**Table 1:** Comparison of socio-demographic variables between two groups p <0.05 is significant. PRP: platelet-rich plasma; DM: diabetes mellitus; IHD: ischemic heart disease; PAD: peripheral arterial disease; COPD: chronic obstructive pulmonary disease

	PRP with conservative management (n=75)	Only conservative management (n=73)	p-value
Age	38.99±7.86	39.21±7.59	0.291
Male: Female	16:59	17:56	0.775
DM	19(25.3%)	21(28.8%)	0.638
IHD	7(9.3%)	9(12.3%)	0.557
PAD	5(6.7%)	6(8.2%)	0.754
Renal impairment	1(1.3%)	2(2.7%)	0.543
COPD	3(4%)	5(6.8%)	0.443
Smoker	31/42	33/41	0.634

**Table 2:** Foot function index (FFI) between two groups P-value reached from Kruskal–Wallis test; p <0.05 is considered significant. PRP: platelet-rich plasma

FFI	PRP with conservative management (n=75)			Only conservative management (n=73)			p-value
	Pain	Disability	Activity limitation	Pain	Disability	Activity limitation	
Baseline	46.30 ±6.14	59.13 ±6.98	7.47 ±1.04	44.63±4.37	56.87 ±4.52	6.70 ±0.59	0.945
4^th^ week	30.73 ±5.12	39.67 ±6.17	5.33 ±1.12	31.90 ±4.29	42.10 ±5.35	4.57 ±0.63	0.716
12^th^ week	17.43±5.10	23.30 ±6.01	2.70 ±1.26	20.30 ±4.10	26.63 ±6.31	2.27 ±0.64	0.628
24^th^ week	7.37±3.10	9.63 ±4.43	0.67 ±0.61	11.90±3.03	16.07 ±4.77	1.10 ±0.40	0.527

Table 3 compares the study population's FFI and VAS scoring. We observed that the FFI scores decreased significantly in group A after a single-dose PRP injection, compared with group B treated with conventional therapy. In group A, FFI scores decreased from 49.09±5.72 to 7.67±3.41. The current study also revealed a statistically significant difference between groups A and B in the 12th week (p=0.04) and 24th week (p=<0.001) regarding the improvement of the FFI scores.

**Table 3:** Comparison of FFI total scores between two study groups FFI: foot function index; VAS: visual analog scale P-value reached from the chi-square test; A p <0.05 is considered significant.

Parameter	PRP with conservative management (n=75)	Only conservative management (n=73)	p-value
FFI			
Baseline	49.09 ±5.72	46.88±3.62	0.078
4^th^ week	32.86 ±5.01	34.13 ±4.12	0.260
12^th^ week	18.92 ±5.27	21.72 ±5.07	0.040
24^th^ week	7.67 ±3.41	12.54 ±3.40	<0.001
VAS			
Baseline	7.27±0.69	7.30±0.65	0.848
4^th^ week	5.07±0.64	5.43±0.63	0.029
12^th^ week	3.00±0.64	3.60±0.72	0.001
24^th^ week	1.47 ±0.51	2.27 ±0.58	<0.001

Furthermore, initially, both groups had a pain score of >7 on the VAS scale (Table 3), but the mean pain score significantly reduced to 1.47±0.51 after six months of single-dose PRP (p<0.001) in group A compared to group B. However, the group B population also experienced a substantial decrease in the VAS scores after conservative treatment alone. Although both study groups observed reduced pain, the PRP group experienced significantly reduced pain in the 4th week, 12th week, and 24th week in contrast to only conservative management.

## Discussion

This quasi-experimental study observed the effect of ultrasound-guided, single-dose PRP injection in a patient with chronic plantar fasciitis among the Bangladeshi population. This study found that FFI scores decreased from 49.09±5.72 to 7.67±3.41 among PRP with the conservative management population. It was observed that there was a statistically significant difference between groups A and B in the 12th week and 24th week regarding FFI scores. In group A, the mean pain score significantly reduced to 1.47±0.51 after six months of single-dose PRP (p<0.001).

This study shows the average age was 38.99±7.86 in group A and 39.21±7.59 in group B. It was observed that 19 patients were DM in group A and 21 patients in group B. A total of seven patients had ischemic heart disease (IHD) in group A and nine patients in group B, five patients had peripheral arterial disease (PAD) in group B. Thirty-one patients had a smoker in group A, and 33 patients had a smoker in the group. The difference was statistically insignificant between the two groups (P>0.05). These findings consisted of several studies [6,7,15-18].

Plantar fasciitis (PF) is a typical lesion in the heel, and approximately 11% to 15% of adult foot symptoms require professional care [16]. Numerous therapies have been reported, but the evidence supporting a preferred treatment needs to be more adequate or consistent [17]. Other studies [8,18-20] also found similar results. A study on 25 plantar fasciitis patients treated with PRP reported that PRP significantly reduced pain [15]. A single-center, uncontrolled, prospective, preliminary study found that VAS for pain significantly decreased from 7.1±1.1 before treatment to 1.9±1.5 at the last follow-up (p<0.01) [18]. In a study, it was observed that there was a significant improve­ment in the visual analog scale score in patients treated with the platelet-rich plas­ma at three weeks and three-months follow-ups [19]. Another study was conducted to find out the efficacy of PRP in relieving pain, where they found PRP to be safe and effective in reducing pain, similar to our study findings [20].

Furthermore, the PRP injection into the affected tissues augments the healing process, which is necessary to reverse the degenerative process in the base of the plantar fascia [21]. Cytokines in platelet granules were found to affect the healing process necessary to reverse chronic plantar fasciitis. Furthermore, transforming growth factor β1 greatly raises type I collagen production by tendon sheath fibroblast, and cytokines have been thought to work dose-dependently [22].

Strengthening exercise is essential in treating plantar fasciitis and correcting functional risk factors such as improved weakness of intrinsic foot muscles. Plantar fasciitis is often attributable to poor intrinsic muscle strength and poor force attenuation [23]. Studies supporting the use of plantar fascia-specific stretching exercises as a critical component of treatment for plantar fasciitis are also found [24]. Stretching reduces the tension in the fascia, which becomes tight during plantar fasciitis. Though both study groups experienced reduced pain, the group A population reduced pain significantly more than group B at the 4th week, 12th week, and 24th-week follow-up. A study conducted on a randomized trial to evaluate the efficacy of PRP compared with conventional treatments for plantar fasciitis found that treatment of plantar fasciitis with PRP resulted in improved pain and functional outcomes compared with conventional therapy alone [10] This might be due to the cellular and humeral mediators of PRP, which provides conditions favorable for tissue healing [23-25].

We observed that the FFI scores decreased significantly after a single dose PRP injection, which was consistent with other studies [26,27]. Nevertheless, Chew et al. compared the functional outcome between the PRP group and conventional group with the American Orthopaedic Foot and Ankle Society (AOFAS) ankle-hind foot scale, where they also found that PRP injection significantly improved functional impairment compared to the conventional group [13]. It was mentionable that no patient developed any adverse reaction to PRP within the study period. This might be due to the advantages of autologous PRP [7].

Being a quasi-experimental nature study, we need to acknowledge a few limitations. The patient-oriented selection of a comparison group might lead to bias, and we cannot exclude other potential factors that influence study outcomes. However, proper clinical assessment and patient counseling prior to group allocation mitigate the selection and outcome bias. Furthermore, the lack of randomization or blind study group could lead to outcome bias. Albeit, study results show significant findings, this small sample represents one megacity in Bangladesh, so study results need to be generalized worldwide.

## Conclusions

This study shows that the early outcome of the single-dose, ultrasound-guided PRP injection in combination with conservative management is more effective than conservative therapy alone for chronic plantar fasciitis based on the VAS and FFI scoring system. In addition to conservative management, we conclude that local PRP injection is a safe and feasible treatment option and carries better outcomes for chronic plantar fasciitis. Although no significant complications and adverse effects were reported during the injection or follow-up period, we recommend future studies, especially enough powered RCTs with a large sample, must determine the safety and efficacy of intra-lesional autologous PRP injection prior to generalized use.
